# Synthetic Fosmidomycin Analogues with Altered Chelating Moieties Do Not Inhibit 1-Deoxy-d-xylulose 5-phosphate Reductoisomerase or *Plasmodium falciparum* Growth *In Vitro*

**DOI:** 10.3390/molecules19022571

**Published:** 2014-02-24

**Authors:** René Chofor, Martijn D.P. Risseeuw, Jenny Pouyez, Chinchu Johny, Johan Wouters, Cynthia S. Dowd, Robin D. Couch, Serge Van Calenbergh

**Affiliations:** 1 Laboratory for Medicinal Chemistry, Ghent University, Harelbekestraat 72, Ghent B-9000, Belgium; E-Mails: rene.chofor@ugent.be (R.C.); martijn.risseeuw@ugent.be (M.D.P.R.); 2 Department of Chemistry, University of Namur, UNamur, Rue de Bruxelles 61, Namur B-5000, Belgium; E-Mails: jenny.pouyez@unamur.be (J.P.); johan.wouters@unamur.be (J.W.); 3 Department of Chemistry and Biochemistry, George Mason University, Manassas, VA 20110, USA; E-Mails: cjohny@gmu.edu (C.J.); rcouch@gmu.edu (R.D.C.); 4 Department of Chemistry, George Washington University, Washington, DC 20052, USA; E-Mail: cdowd@gwu.edu

**Keywords:** fosmidomycin, DOXP reductoisomerase, non-mevalonate pathway, isoprenoid biosynthesis, coordination chemistry

## Abstract

Fourteen new fosmidomycin analogues with altered metal chelating groups were prepared and evaluated for inhibition of *E. coli* Dxr, *M. tuberculosis* Dxr and the growth of *P. falciparum* K1 in human erythrocytes. None of the synthesized compounds showed activity against either enzyme or the *Plasmodia*. This study further underlines the importance of the hydroxamate functionality and illustrates that identifying effective alternative bidentate ligands for this target enzyme is challenging.

## 1. Introduction

Yearly, up to 5 million clinical cases and a million fatalities result from malaria, an infectious disease caused by protozoa of the *Plasmodium* species, with *P. falciparum* being responsible for the most severe cases [1]. The heaviest caseload is suffered by pregnant women and children in sub-Saharan Africa [2]. Unlike *Plasmodia* which are endemic in the tropics, *Mycobacterium tuberculosis* (Mtb), the causative agent of tuberculosis afflicts one-third of the world’s population annually, leading to about 2–3 million deaths [3]. With resistance emerging to virtually all currently used drugs for the treatment of both diseases, new, safe, effective and low cost antimalarial and antitubercular therapeutics are highly awaited. 

**Figure 1:**
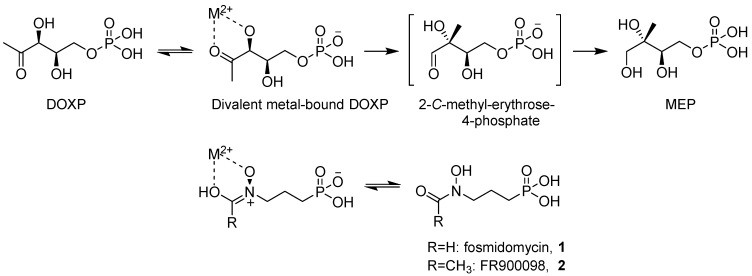
Analogy between DOXP and Fosmidomycin/FR900098.

The discovery that fosmidomycin (**1**, Figure 1) and its acetyl congener FR900098 (**2**), both natural products extracted from *Streptomyces* species inhibit 1-deoxy-d-xylulose-5-phosphate reducto-isomerase (Dxr), opened interesting opportunities for therapeutics [4,5]. Dxr is the second enzyme in the non-mevalonate pathway (NMP) for isoprenoid biosynthesis, which is absent in humans, but present in most Gram-negative and some Gram-positive bacteria (including Mtb), as well as in apicomplexan parasites (including *Plasmodia*) [6,7]. Fosmidomycin inhibits the Dxr-catalyzed conversion of 1-deoxy-d-xylulose-5-phosphate (DOXP) to 2*C*-methyl-d-erythritol-4-phosphate (MEP), by mimicking the binding mode of DOXP to this enzyme [8,9]. SAR studies have indicated the importance of fosmidomycin’s hydroxamate moiety for chelation of a divalent metal cation (M: Mn^2+^ or Mg^2+^) present in the enzyme’s active site.

Due to its promising antimalarial activity, fosmidomycin received considerable attention and a combination therapy with clindamycin confirmed its potential as an antimalarial drug, following clinical trials conducted in Gabon and Thailand [10,11]. However, the moderate bioavailability and short serum half-life of fosmidomycin prevented the drug combination from reaching the market. Fosmidomycin’s phosphonate group is highly ionized at physiological pH, which is the main reason for its low bioavailability. While this does not preclude efficient uptake in *P. falciparum*, other organisms like Mtb, are not sensitive to fosmidomycin because they lack a glycerol-3-phosphate transporter (G1pT) that is known to actively transport fosmidomycin across hydrophobic cell membranes [12,13]. 

Although the chelating ability of hydroxamates often makes them potent metalloenzyme inhibitors, most hydroxamic acids suffer from poor oral bioavailability and significant binding to other metals (e.g., Zn^2+^, Cu^2+^, *etc.*) besides Mn^2+^ and Mg^2+^ [14,15]. In addition, hydroxamic acids may be rapidly degraded *in vivo* by hydrolysis, glucuronidation and sulfation and may suffer from poor pharmacokinetic and toxicological profiles [16]. In order to circumvent the limitations associated with the phosphonate and hydroxamate moiety of fosmidomycin, two strategies have been widely exploited in the design of potent analogues: masking of the polar phosphonate group as prodrugs and/or substituting the hydroxamate of fosmidomycin with an alternative Mn^2+^ and Mg^2+^ binding group. The former strategy has been relatively well investigated [17], while the latter has been studied with less rigor.

**Figure 2:**
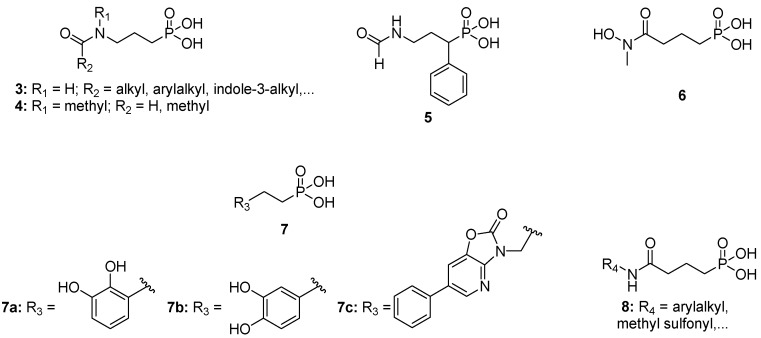
Hydroxamate-modified analogs of fosmidomycin.

Giessmann *et al.* synthesized a series of amidopropylphosphonates **3** (Figure 2), but none of these showed detectable *E. coli* Dxr inhibition when tested up to 30 µM, indicating the importance of the N-OH group for Dxr inhibition [18]. This was further proven by Woo *et al.* following the evaluation of compounds **4** wherein the N-OH was replaced with N-CH_3_ [19]. During the synthesis of α-substituted fosmidomycin analogues, our group observed that benzyl removal from the retrohydroxamate moiety by catalytic hydrogenation typically resulted in the formation of the desired compound, but also significant amounts of the corresponding deoxygenated derivative, i.e., the amide, due to the competitive side reaction of “full” reduction [20]. Deprotection of the phosphonate moiety of the latter afforded analogues such as **5**, which were moderately potent in inhibiting *E. coli* Dxr and capable of inhibiting the growth of a Dd2 *P. falciparum* strain at submicromolar concentrations (unpublished results). The Rohmer group demonstrated that the reverse hydroxamate counterparts of fosmidomycin or FR900098 (**6**) elicit comparable inhibitory activity against *E. coli* Dxr as the natural products [21]. This observation was further confirmed by other groups which obtained sub-micromolar IC50 values following evaluation of fosmidomycin analogues comprising a reverse hydroxamate moiety [22,23,24]. Nakamura and co-workers showed that a *cis* arrangement of the two oxygen atoms of the hydroxamate group is required for effective metal chelation. Furthermore, they suggested that alternative functional groups containing *cis* oxygen atoms might have comparable metal coordination ability [8]. Catechols **7a** and **7b** showed IC_50_ values of 24.8 µM and 4.5 µM, respectively, when tested for inhibition of *E. coli* Dxr, indicating a preference for the 1,3,4-orientation (**7b**) of the catechol over the 1,2,3-orientation (**7a**) [25].

In search for lipophilic fosmidomycin analogues, Andaloussi *et al.* resynthesized **7b** alongside other hydroxamate-modified compounds with a bulky heteroaryl moiety such as **7c**. Tests conducted with these compounds revealed that steric constraint in the vicinity of the Dxr active site was deleterious to inhibitory potency [26]. Other attempts to substitute the hydroxamate group of fosmidomycin with similar sterically demanding alternatives led to the conclusion that the Dxr active site is very narrow around the metal cation [27,28]. Nevertheless, the Dowd group recently observed a more efficient coordination of the metal cation by amide- *versus* *O*-linked substituents on the retrohydroxamate of fosmidomycin [29]. They highlighted the importance of having an aromatic group in the inhibitor while also suggesting that an alkyl chain between the retrohydroxamate and the aryl group may be preferable for accessing an alternate binding location. 

This paper aims to more systematically investigate the possibilities of replacing the retrohydroxamate group of fosmidomycin with effective alternative bidentate ligands. Amide derivatives represented by the general structure **8** were prepared and evaluated. We envisaged a contribution to chelation by *ortho*-substituents on the amide-linked aromatic ring. Compounds with a NH moiety between carbonyl and sulfonyl groups are very acidic (pKa ~ 2). At physiological pH, the presence of a negative charge at this position would be expected to improve the interaction with the active-site metal ion [30]. Therefore, we included one analogue with a methylsulfonyl group in the *ortho* position of the phenyl ring (compound **8h**), as well as a (non-aromatic) sulfamate (compound **8m**). In order to ascertain the influence of electronic factors on chelation, aromatic substituents with various electronic properties were selected.

## 2. Results and Discussion

### 2.1. Synthesis

**Scheme 1:**
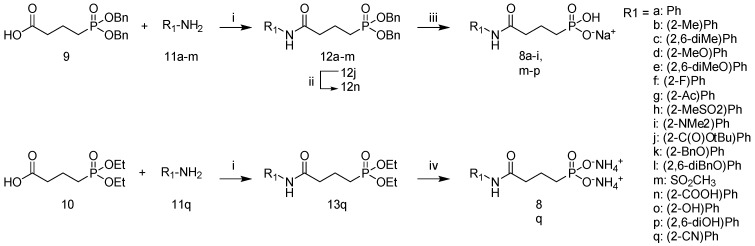
General synthesis of amide derivatives **8a**–**i**, **m**–**q.**

**Scheme 2:**
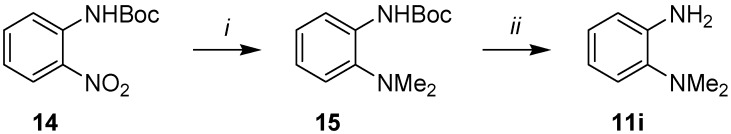
Preparation of aniline **11i**.

The synthesis of the amide derivatives **8a**–**i**, **m**–**q** is outlined in Scheme 1. Carboxylic acid **9** was readily prepared starting from commercially available ethyl 4-bromo-butyrate and dibenzyl phosphite as previously described by Kuntz *et al.* [21]. Anticipation that the cyano substituent on aniline **11q** would be susceptible to hydrogenation later in the synthesis necessitated the use of the diethyl protected phosphonate **10**, obtained from saponification of commercially available triethyl 4-phosphonobutyrate, for reaction with this aniline. With the exception of anilines **11i** and **11l**, all other anilines used were commercially available. Synthesis of **11i** (Scheme 2) started from 2-nitro-aniline which was easily converted to the NH-Boc protected form as described by McNeil and Kelly [31]. Subsequent *N*, *N*-dimethylation, followed by Boc removal afforded the aniline. Compound **11l** was prepared from 2,6-dihydroxyaniline according to a literature procedure [32].

Anilines are often poor nucleophiles, thus carboxylic acids **9** and **10** were first converted to their respective acid chlorides by treatment with oxalyl chloride before subsequent nucleophilic substitution of **11a**–**m**, **11q** to generate a small library of the protected amides **12a**–**m**, and **13q** in moderate yields. The ^1^H-NMR spectrum of **12c** displays two peaks at 2.17 ppm and 2.21 ppm for the 2,6-dimethyl protons corresponding to the *E* and *Z* amide rotamers in a 5/1 ratio. Hydrolysis of the tertiary butyl ester group of **12j** with TFA (20% in dichloromethane) further converted this intermediate to **12n**. Using benzyl protection for both the phosphonate and the aryl substituent (**12k** and **12l**) allowed a mild single deprotection by catalytic hydrogenolysis in the presence of palladium over activated charcoal at room temperature to access targets **8a**–**i**, **m**–**p**. TMSBr mediated deprotection of **13q** and basic workup yielded **8q** as the bisammonium salt. 

*Reagents and conditions*: (i) oxalyl chloride, DMF, CH_2_Cl_2_, 45 °C, 2–3 h, 40%–75%; (ii) TFA/CH_2_Cl_2_ (for **12j**); (iii) H_2_, Pd/C, MeOH, NaOHaq., 25 °C, 10–15 min, quant.; (iv) TMSBr, CH_2_Cl_2_, H_2_O, NH_4_OHaq., quant.

*Reagents and conditions*: (i) formaldehyde, H_2_, Pd/C, MeOH, 90%; (ii) acetyl chloride, MeOH.

### 2.2. Antiplasmodial and Antitubercular Evaluation

**Figure 3:**
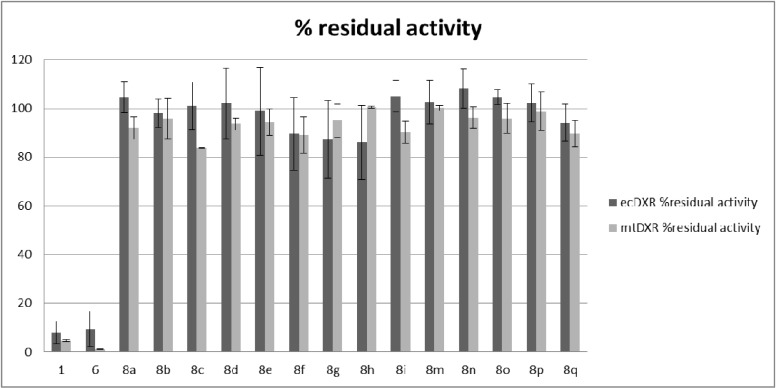
Relative activity of **8a**–**i, m**–**q** on purified *E. coli* (dark grey) and Mtb Dxr (light-grey).

The ability of the final compounds to inhibit the *E. coli* Dxr and *M. tuberculosis* Dxr was investigated using a spectrophotometric assay monitoring the substrate dependent oxidation of NADPH, essentially as described in detail elsewhere [33]. As shown in Figure 3, at a concentration of 100 µM, all compounds failed to significantly inhibit the *E. coli* or Mtb Dxr. Likewise all compounds were found essentially inactive against *P. falciparum* K1 in human erythrocytes (IC_50_ > 64 µM).

Similar to fosmidomycin, we expected that the phosphonate group of these analogs would be accommodated in the phosphate binding pocket of Dxr. With the three-carbon spacer unaltered, the introduced modification of the hydroxamate group is determining the lack of Dxr inhibitory activity. Monodentate ligands include virtually all anions and simple Lewis bases. While anticipating that the bivalent metal cation would be more readily bound by electron rich substituents on the aromatic ring, we expected that the analogs with 2,6-disubstituted aromatic rings would elicit better enzyme inhibition than their monosubstituted counterparts, since possible rotation of the amide bond would still assure a favorable conformation (*cis*) with respect to the carbonyl oxygen. Even though the hard metal ion character of Mg^2+^ favors the formation of stable complexes with dioxygen based hard ligands, *O*-linked substituents on the ring did not improve the inhibitory ability of these analogues. Carboxylate is a known chelating group [34] but in the assay conditions, the group was possibly protonated thereby reducing the chelating potency of the carboxylate oxygen of **8n** with the Mg^2+^ ion. Obviously, the presence of an aromatic ring improved the lipophilicity of these analogs. However, limited flexibility around the amide bond seems detrimental for inhibitory activity. Maybe, the introduction of methylene groups between the NH and the (substituted)phenyl ring could increase the likelihood of adopting of a better conformation for occupation of ‘alternative’ binding pockets or a better fitting of the compound into the active site. In the course of our work, Bodill *et al.* reported similar modifications of the reytrohydroxamate moiety of fosmidomycin [35]. Out of a series of phosphonated *N*-(hetero)arylcarboxamide analogues with one, two, three or four methylene groups linking the phosphonate to the carboxamide group, they found that increasing the number of methylene groups in the spacer (particularly to three or four methylene groups) decreases the Dxr inhibitory activity dramatically. The authors noted that while receptor-cavity size constraints is an important determinant of binding, allosteric and reverse-orientation ligand binding modes cannot be excluded. 

## 3. Experimental

### 3.1. General Methods and Materials

^1^H-, ^13^C-, ^19^F- and ^31^P-NMR spectra were recorded in CDCl_3_, or D_2_O on a Mercury 300 spectrometer (Varian, Palo Alto, CA, USA). Chemical shifts are given in parts per million (ppm) (δ relative to TMS for H and C and to external D_3_PO_4_ for ^31^P. High resolution mass spectroscopy spectra for all compounds were also recorded on a LCT Premier XE orthogonal time-of flight spectrometer with API-ES source (Waters, Alliance 2695XE-LCT Premier XE^TM^, Zellik, Belgium). Silica gel (60 Å, 0.063–0.200 mm) was purchased from Biosolve (Valkenswaard, The Netherlands). All solvents and chemicals were used as purchased unless otherwise stated. 

### 3.2. General Procedure for the Synthesis of Protected Amides

To a 0.5 M solution of the acid **9/10** in dichloromethane under nitrogen atmosphere, was added oxalyl chloride (2 eq.) and a few drops of DMF at room temperature. After effervescence subsided, the mixture was heated to reflux at 45 °C for 2 h. It was then cooled to room temperature, concentrated *in vacuo*, co-evaporated three times with toluene and then re-dissolved in dichloromethane. The aniline (2 eq.) was then added at 0 °C, followed by DIPEA (3 eq.) and the mixture stirred overnight at room temperature. The reaction was quenched by addition of NaHCO_3_ and the aqueous layer was extracted three times with dichloromethane. The combined organic layer was washed once with brine, dried over Na_2_SO_4_ and concentrated *in vacuo*. Purification by silica gel chromatography using a toluene/acetone or dichloromethane/methanol solvent system gave access to the pure protected amides (30%–75% yields).

*Dibenzyl 3-(phenylcarbamoyl)propylphosphonate* (**12a**). ^1^H-NMR (300 MHz, CDCl_3_) δ_H_ ppm 1.55–2.09 (m, 4H, P-CH_2_-CH_2_), 2.47 (t, *J* = 6.82 Hz, 2H, CH_2_-CONHPh), 4.89–5.11 (m, 4H, CH_2_-Ph), 6.99–7.55 (m, 15H, Ar-H), 8.27 (br. s, 1H, NH). ^13^C-NMR (75 MHz, CDCl_3_) δ_C_ ppm 18.93 (d, ^2^*J*_P-C_ = 6.32 Hz, C2), 24.42 (d, ^1^*J*_P-C_ = 139.32 Hz, C1), 36.82 (d, ^3^*J*_P-C_ = 8.85 Hz), 67.39 (d, ^2^*J*_P-C_ = 6.63 Hz, PhCH_2,_ C3), 119.77 (Ar-C), 119.87 (Ar-C), 124.23 (Ar-C), 128.22 (Ar-C), 128.80 (Ar-C), 136.12 (^3^*J*_P-C_ = 5.53 Hz, C_ipso_-PhCH_2_), 136.31, (Ar-C) 138.39 (Ar-C), 170.71 (CO). ^31^P-NMR (121.5 MHz, CDCl_3_): δ_P_ppm = 34.00. HRMS (ESI): calculated for C_24_H_27_NO_4_P [(M+H)^+^], 424.1672; found 424.1698.

*Dibenzyl 3-(o-tolylcarbamoyl)propylphosphonate* (**12b**). ^1^H-NMR (300 MHz, CDCl_3_) δ_H_ ppm 1.70–2.12 (m, 4H, P-CH_2_-CH_2_), 2.24 (s, Ph-CH_3_), 2.51 (t, *J* = 6.74 Hz, 2H, CH_2_-CONHPh), 4.87–5.12 (m, 4H, CH_2_-Ph), 7.00–7.23 (m, 3H), 7.28–7.38 (m, 10H, Ar-H), 7.55 (br. s, 1H, NH), 7.78 (d, *J* = 7.91 Hz, 1H, Ar-H). ^13^C-NMR (75 MHz, CDCl_3_) δ_C_ ppm 18.18 (PhCH_3_), 19.16 (d, ^2^*J*_P-C_ = 6.32 Hz, C2), 24.75 (d, ^1^*J*_P-C_ = 140.21 Hz, C1), 36.87 (d, ^3^*J*_P-C_ = 9.34 Hz, C3), 67.52 (^2^*J*_P-C_ = 6.54 Hz, PhCH_2_), 123.35 (Ar-C), 125.32 (Ar-C), 126.85 (Ar-C), 128.18 (Ar-C), 128.74 (Ar-C), 128.86 (Ar-C), 130.70 (Ar-C), 136.00 (Ar-C), 136.43 (d, ^3^*J*_P-C_ = 5.93 Hz, C_ipso_-PhCH_2_), 170.70 (CO). ^31^P-NMR (121.5 MHz, CDCl_3_): δ_P_ ppm = 33.77. HRMS (ESI): calculated for C_25_H_29_NO_4_P [(M+H)^+^], 438.1829; found 438.1831.

*Dibenzyl 3-(2,6-dimethylphenylcarbamoyl)propylphosphonate* (**12c**). ^1^H-NMR (300 MHz, CDCl_3_) δ_H_ ppm 1.69–2.12 (m, 4H, P-CH_2_-CH_2_), 2.17 (5/6 of 6H, s, Ph-CH_3_), 2.17 (1/6 of 6H, s, Ph-CH_3_), 2.49 (t, *J* = 7.16 Hz, 2H, CH_2_-CONHPh), 4.86–5.14 (m, 4H, CH_2_-Ph), 7.02–7.14 (m, 3H, Ar-H), 7.29–7.38 (m, 10H, Ar-H). ^13^C-NMR (75 MHz, CDCl_3_) δ_C_ ppm 18.79 (Ph-CH_3_), 19.22 (d, ^2^*J*_P-C_ = 5.21 Hz, C2), 25.20 (d, ^1^*J*_P-C_ = 140.50 Hz, C1), 36.24 (d, ^3^*J*_P-C_ = 10.92 Hz, C3), 67.49 (d, ^2^*J*_P-C_ = 6.69 Hz, PhCH_2_), 127.47 (Ar-C), 128.28 (Ar-C), 128.38 (Ar-C), 128.74 (Ar-C), 128.87 (Ar-C), 134.14 (Ar-C), 135.53 (Ar-C), 136.48 (d, ^3^*J*_P-C_ = 5.85 Hz, C_ipso_-PhCH_2_), 170.57 (CO). ^31^P-NMR (121.5 MHz, CDCl_3_): δ_P_ ppm = 33.63. HRMS (ESI): calculated for C_26_H_31_NO_4_P [(M+H)^+^], 452.1985; found 452.1990.

*Dibenzyl 3-(2-methoxyphenylcarbamoyl)propylphosphonate* (**12d**). ^1^H-NMR (300 MHz, CDCl_3_) δ_H_ ppm 1.74–2.12 (m, 4H, P-CH_2_-CH_2_), 2.47 (t, *J* = 7.04 Hz, 2H, CH_2_-CONHPh), 3.83 (s, 3H, NHPh-O-CH_3_), 4.91–5.10 (m, 4H, CH_2_-Ph), 6.86 (dd, *J* = 1.17 Hz, 7.91 Hz, 1H, Ar-H), 6.94 (td, *J* = 1.46 Hz, 7.61 Hz, 1H, Ar-H), 7.03 (td, *J* = 1.76 Hz, 7.62 Hz), 7.26–7.40 (m, 10H, Ar-H), 7.82 (br. s, 1H, NH), 8.33 (dd, *J* = 1.17 Hz, 7.91 Hz, 1H, Ar-H). ^13^C-NMR (75 MHz, CDCl_3_) δ_C_ ppm 18.61 (d, ^2^*J*_P-C_ = 4.98 Hz, C2), 25.09 (d, ^1^*J*_P-C_ = 140.42 Hz, C1), 37.48 (d, ^3^*J*_P-C_ = 13.27 Hz, C3), 55.67 (Ph-O-CH_3_) 67.23 (^2^*J*_P-C_ = 6.64 Hz, PhCH_2_), 109.97 (Ar-C), 119.98 (Ar-C), 121.08 (Ar-C), 123.76 (Ar-C), 127.61 (Ar-C), 127.99 (Ar-C), 128.46 (Ar-C), 128.65 (Ar-C), 136.41 (d, ^3^*J*_P-C_ = 6.08 Hz, C_ipso_-PhCH_2_), 147.889 (Ar-C), 170.03 (CO). ^31^P-NMR (121.5 MHz, CDCl_3_): δ_P_ ppm = 33.52. HRMS (ESI): calculated for C_25_H_29_NO_5_P [(M+H)^+^], 454.1778; found 454.1791.

*Dibenzyl 3-(2,6-dimethoxyphenylcarbamoyl)propylphosphonate* (**12e**). ^1^H-NMR (300 MHz, CDCl_3_) δ_H_ ppm 1.85–2.11 (m, 4H, P-CH_2_-CH_2_), 2.33–2.59 (m, 2H, CH_2_-CONHPh), 3.75 (br. s, 6H, OCH_3_) 4.86–5.12 (m, 4H, CH_2_-Ph), 6.55 (d, *J* = 8.51 Hz, 2H, Ar-H), 7.17 (t, *J* = 8.52 Hz, 1H, Ar-H) 7.27–7.36 (m, 10H, Ar-H). ^13^C-NMR (75 MHz, CDCl_3_) δ_C_ ppm 18.12 (Ph-CH_3_), 22.26 (d, ^2^*J*_P-C_ = 5.35 Hz, C2), 25.28 (d, ^1^*J*_P-C_ = 139.10 Hz, C1), 36.21 (d, ^3^*J*_P-C_ = 9.83 Hz, C3), 67.38 (d, ^2^*J*_P-C_ = 6.58 Hz, PhCH_2_), 127.39 (Ar-C), 128.20 (Ar-C), 128.32 (Ar-C), 128.45 (Ar-C), 128.92 (Ar-C), 129.13 (Ar-C), 135.51 (Ar-C), 136.97 (d, ^3^*J*_P-C_ = 6.08 Hz, C_ipso_-PhCH_2_), 165.22 (CO). ^31^P-NMR (121.5 MHz, CDCl_3_): δ_P_ ppm = 33.07. HRMS (ESI): calculated for C_26_H_31_NO_6_P [(M+H)^+^], 484.1884 ; found 484.0402.

*Dibenzyl 3-(2-fluorophenylcarbamoyl)propylphosphonate* (**12f**). ^1^H-NMR (300 MHz, CDCl_3_) δ_H_ ppm 1.76–2.01 (m, 4H, P-CH_2_-CH_2_), 2.50 (t, *J* = 7.06 Hz, 2H, CH_2_-CONHPh), 4.88–5.15 (m, 4H, CH_2_-Ph), 6.96–7.16 (m, 3H, Ar-H), 7.28–7.39 (m, 10H, Ar-H), 7.84 (br. s, 1H, NH), 8.25 (t, *J* = 8.18 Hz, 1H, Ar-H). ^13^C-NMR (75 MHz, CDCl_3_) δ_C_ ppm 18.64 (d, ^2^*J*_P-C_ = 5.24 Hz, C2), 24.71 (d, ^1^*J*_P-C_ = 140.37 Hz, C1), 36.85 (d, ^3^*J*_P-C_ = 10.64 Hz, C3), 67.29 (d, ^2^*J*_P-C_ = 6.59 Hz, PhCH_2_), 114.86 (d, ^2^*J*_F-C_ = 19.38 Hz, F-Ph), 122.02 (Ar-C), 124.36 (d, ^2^*J*_F-C_ = 7.58 Hz, F-Ph), 124.49 (d, ^3^*J*_F-C_ = 3.79 Hz, F-Ph), 128.00 (Ar-C), 128.49 (Ar-C), 128.62 (Ar-C), 136.26 (d, ^3^*J*_P-C_ = 5.71 Hz, C_ipso_-PhCH_2_), 152.42 (d, ^1^*J*_F-C_ = 243.71 Hz, F-Ph), 170.43 (CO). ^31^P-NMR (121.5 MHz, CDCl_3_): δ_P_ ppm = 33.60. HRMS (ESI): calculated for C_24_H_26_FNO_4_P [(M+H)^+^], 442.1578; found 442.1586.

*Dibenzyl 3-(2-acetylphenylcarbamoyl)propylphosphonate* (**12g**). ^1^H-NMR (300 MHz, CDCl_3_) δ_H_ ppm 1.80–2.12 (m, 4H, P-CH_2_-CH_2_), 2.49 (t, *J* = 7.11 Hz, 2H, CH_2_-CONHPh), 2.65 (s, 3H, OCCH_3_), 4.93–5.11 (m, 4H, CH_2_-Ph), 7.11 (dd, 1H, *J* = 1.17 Hz, 8.23 Hz, Ar-H), 7.29–7.38 (m, 10H, Ar-H), 7.54 (dd, *J* = 1.68 Hz, 8.52 Hz, 1H, Ar-H), 7.88 (dd, *J* = 1.50 Hz, 7.86 Hz, 1H, Ar-H), 8.72 (dd, *J* = 1.10 Hz, 8.52, 1H Ar-H), 11.70 (br. s, 1H, NH). ^13^C-NMR (75 MHz, CDCl_3_) δ_C_ ppm 18.59 (d, ^2^*J*_P-C_ = 4.42 Hz, C2), 25.45 (d, ^1^*J*_P-C_ = 140.98 Hz, C1), 28.69 (PhCOCH_3_), 38.45 (d, ^3^*J*_P-C_ = 16.03 Hz, C3), 67.25 (d, ^2^*J*_P-C_ = 6.63 Hz, PhCH_2_), 120.82 (Ar-C), 121.90 (Ar-C), 122.45 (Ar-C), 128.01 (Ar-C), 128.45 (Ar-C), 128.70 (Ar-C), 131.79 (Ar-C), 135.30 (Ar-C), 136.45 (d, ^3^*J*_P-C_ = 6.08 Hz, C_ipso_-PhCH_2_), 141.07 (Ar-C), 174.24 (CO), 202.91 (PhCOCH_3_). ^31^P-NMR (121.5 MHz, CDCl_3_): δ_P_ ppm = 33.43. HRMS (ESI): calculated for C_26_H_29_NO_5_P [(M+H)^+^], 466.1778; found 466.1779. 

*Dibenzyl 3-(2-(methylsulfonyl)phenylcarbamoyl)propylphosphonate* (**12h**). ^1^H-NMR (300 MHz, CDCl_3_) δ_H_ ppm 1.74–2.12 (m, 4H, P-CH_2_-CH_2_), 2.50 (t, *J* = 7.10 Hz, 2H, CH_2_-CONHPh), 2.99 (br. s, 3H, SO_2_-CH_3_), 4.92–5.11 (m, 4H, CH_2_-Ph), 7.21–7.29 (m, 2H, Ar-H), 7.30–7.37 (m, 10H, Ar-H), 7.62 (td, *J* = 1.62 Hz, 7.07 Hz, 1H, Ar-H), 7.90 (dd, *J* = 1.62 Hz, 7.98 Hz), 8.45 (dd, *J* = 1.27 Hz, 8.01 Hz, 1H, Ar-H). ^13^C-NMR (75 MHz, CDCl_3_) δ_C_ ppm 18.56 (d, ^2^*J*_P-C_ = 5.07 Hz, C2), 25.42 (d, ^1^*J*_P-C_ = 141.36 Hz, C1), 37.92 (d, ^3^*J*_P-C_ = 14.76 Hz, C3), 44.41 (-PhSO_2_CH_3_), 67.47 (d, ^2^*J*_P-C_ = 6.82 Hz, PhCH_2_), 123.06 (Ar-C), 124.40 (Ar-C), 127.28 (Ar-C), 128.21 (Ar-C), 128.68 (Ar-C),128.85 (Ar-C), 129.54 (Ar-C), 135.54 (Ar-C), 136.53 (d, ^3^*J*_P-C_ = 5.81 Hz, C_ipso_-PhCH_2_), 137.11 (Ar-C), 170.66 (CO). ^31^P-NMR (121.5 MHz, CDCl_3_): δ_P_ ppm = 33.10. HRMS (ESI): calculated for C_25_H_29_NO_6_PS [(M+H)^+^], 502.1448; found 502.1470. 

*Dibenzyl 3-(2-(dimethylamino)phenylcarbamoyl)propylphosphonate* (**12i**). ^1^H-NMR (300 MHz, CDCl_3_) δ_H_ ppm 1.75–2.12 (m, 4H, P-CH_2_-CH_2_), 2.49 (t, *J* = 7.07 Hz, 2H, CH_2_-CONHPh), 2.60 (br. s, 6H, N-(CH_3_)_2_), 4.92–5.10 (m, 4H, CH_2_-Ph), 7.00–7.18 (m, 3H, Ar-H), 7.27–7.38 (m, 10H, Ar-H), 8.33 (d, 1H, *J* = 7.78, Ar-H), 8.43 (br. s, 1H, NH). ^13^C-NMR (75 MHz, CDCl_3_) δ_C_ ppm 18.80 (d, ^2^*J*_P-C_ = 4.81 Hz, C2), 25.51 (d, ^1^*J*_P-C_ = 140.87 Hz, C1), 37.89 (d, ^3^*J*_P-C_ = 14.19 Hz, C3), 45.00 (N-CH_3_), 67.41 (d, ^2^*J*_P-C_ = 6.60 Hz, PhCH_2_), 119.72 (Ar-C), 120.12 (Ar-C), 123.92 (Ar-C), 125.26 (Ar-C), 128.15 (Ar-C), 128.63 (Ar-C), 128.82 (Ar-C), 133.53 (Ar-C), 136.58 (d, ^3^*J*_P-C_ = 6.02 Hz, C_ipso_-PhCH_2_), 142.87 (Ar-C), 170.16 (CO). ^31^P-NMR (121.5 MHz, CDCl_3_): δ_P_ ppm = 33.53. HRMS (ESI): calculated for C_26_H_32_N_2_O_4_P [(M+H)^+^], 467.2094; found 467.2330. 

*Dibenzyl 3-(2-(tert-butoxycarbonyl)phenylcarbamoyl)propylphosphonate* (**12j**). ^1^H-NMR (300 MHz, CDCl_3_) δ_H_ ppm 1.59 (br. s, 9H, O-*t*Bu), 1.77–2.13 (m, 4H, P-CH_2_-CH_2_), 2.50 (t, *J* = 7.21 Hz, 2H, CH_2_-CONHPh), 4.93–5.11 (m, 4H, CH_2_-Ph), 7.05 (td, *J* = 1.10 Hz, 7.38, 1H, Ar-H), 7.25–7.38 (m, 10H, Ar-H), 7.49 (td, *J* = 1.75 Hz, 7.38 Hz, 1H, Ar-H), 7.97 (dd, *J* = 1.75 Hz, 8.32 Hz 1H, Ar-H), 8.67 (dd, *J* = 1.06 Hz, 8.51 Hz 1H, Ar-H), 11.20 (br. s, 1H, NH). ^13^C-NMR (75 MHz, CDCl_3_) δ_C_ ppm 18.66 (d, ^2^*J*_P-C_ = 4.94 Hz, C2), 25.54 (d, ^1^*J*_P-C_ = 140.62 Hz, C1), 28.41 (PhCOOCCH_3_), 38.55 (d, ^3^*J*_P-C_ = 15.82 Hz, C3), 67.38 (d, ^2^*J*_P-C_ = 6.35 Hz, PhCH_2_), 82.66 (Ar-C), 116.62 (Ar-C), 120.47 (Ar-C), 122.48 (Ar-C), 128.15 (Ar-C), 128.57 (Ar-C), 128.79 (Ar-C), 131.24 (Ar-C), 134.31 (Ar-C), 136.63 (d, ^3^*J*_P-C_ = 5.92 Hz, C_ipso_-PhCH_2_), 141.71 (Ar-C), 167.91 (COO*t*Bu), 170.96 (C0). ^31^P-NMR (121.5 MHz, CDCl_3_): δ_P_ ppm = 33.41. 

*Dibenzyl 3-(2-(benzyloxy)phenylcarbamoyl)propylphosphonate* (**12k**). ^1^H-NMR (300 MHz, CDCl_3_) δ_H_ ppm 1.74–2.10 (m, 4H, P-CH_2_-CH_2_), 2.40 (t, *J* = 7.21 Hz, 2H, CH_2_-CONHPh), 4.86–5.16 (m, 4H, CH_2_-Ph), 5.10 (br. s, 2H, NH-Ph-O-CH_2_-Ph), 6.88–7.05 (m, 3H, Ar-H), 7.24–7.43 (m, 15H, Ar-H), 7.79 (br. s, 1H, NH), 8.35 (td, *J* = 2.47 Hz, 7.84 Hz, 1H, Ar-H). ^13^C-NMR (75 MHz, CDCl_3_) δ_C_ ppm 19.25 (d, ^2^*J*_P-C_ = 4.98 Hz, C2), 25.66 (d, ^1^*J*_P-C_ = 140.43 Hz, C1), 38.00 (d, ^3^*J*_P-C_ = 14.37 Hz, C), 67.54 (^2^*J*_P-C_ = 6.6.63 Hz, PhCH_2_), 71.50 (NH-Ph-O-CH_2_-Ph), 112.35 (Ar-C), 120.72 (Ar-C), 122.04 (Ar-C), 124.28 (Ar-C), 128.09(Ar-C), 128.49(Ar-C), 128.90 (Ar-C), 128.95 (Ar-C), 129.14 (Ar-C), 129.35 (Ar-C), 136.91 (d, ^3^*J*_P-C_ = 6.09 Hz, C_ipso_-PhCH_2_), 136.97 (Ar-C), 147.66 (Ar-C), 170.49 (CO). ^31^P-NMR (121.5 MHz, CDCl_3_): δ_P_ ppm = 33.55. HRMS (ESI): calculated for C_31_H_33_NO_5_P [(M+H)^+^], 530.2091; found 530.2122.

*Dibenzyl 3-(2,6-bis(benzyloxy)phenylcarbamoyl)propylphosphonate* (**12l**). ^1^H-NMR (300 MHz, CDCl_3_) δ_H_ ppm 1.69–1.98 (m, 4H, P-CH_2_-CH_2_), 2.38 (app. s, 2H, CH_2_-CONHPh), 5.82–5.01 (m, 4H, P-O-CH_2_-Ph), 5.06 (s, 4H, N-Ph-O-CH_2_-Ph), 6.62 (d, *J* = 8.57 Hz, 2H, Ar-H), 7.12 (t, *J* = 8.39 Hz, 1H, Ar-H), 7.21–7.45 (m, 20 H, Ar-H). ^13^C-NMR (75 MHz, CDCl_3_) δ_C_ ppm 18.19 (C2), 24.49 (d, ^1^*J*_P-C_ = 138.22 Hz, C1), 36.07 (C3), 67.02 (d, ^2^*J*_P-C_ = 6.59 Hz, PhCH_2_OP), 70.71 (NH-PhOCH_2_Ph), 106.08 (Ar-C), 115.21 (Ar-C), 127.33 (Ar-C), 127.89 (Ar-C), 128.31 (Ar-C), 128.53 (Ar-C), 136.39, (d, ^3^*J*_P-C_ = 5.53 Hz, C_ipso_-PhCH_2_), 138.81 (Ar-C), 154.92 (CO). ^31^P-NMR (121.5 MHz, CDCl_3_): δ_P_ppm = 34.16. 

*Dibenzyl (4-(methylsulfonamido)-4-oxobutyl)phosphonate* (**12m**). ^1^H-NMR (300 MHz, CDCl_3_) δ_H_ ppm 1.75–2.04 (m, 4H, P-CH_2_-CH_2_), 2.47 (t, *J* = 7.03 Hz, 2H, CH_2_-CONHPh), 3.21 (s, 3H, SO_2_NHCH_3_), 4.89–5.11 (m, 4H, CH_2_-Ph), 7.28–7.40 (m, 10H, Ar-H), 10.63 (br.s, 1H, NH). ^13^C-NMR (75 MHz, CDCl_3_) δ_C_ ppm 17.79 (d, ^2^*J*_P-C_ = 5.93 Hz, C2), 24.46 (d, ^1^*J*_P-C_ = 140.57 Hz, C1), 35.84 (d, ^3^*J*_P-C_ = 10.02 Hz, C3), 41.61 (SO_2_NHCH_3_), 67.93 (d, ^2^*J*_P-C_ = 6.47 Hz, PhCH_2_), 128.14 (Ar-C), 128.88 (Ar-C), 128.93 (Ar-C), 136.18 (d, ^3^*J*_P-C_ = 5.81 Hz, C_ipso_-PhCH_2_), 172.17 (CO). ^31^P-NMR (121.5 MHz, CDCl_3_): δ_P_ ppm = 33.41. HRMS (ESI): calculated for C_19_H_25_NO_6_PS [(M+H)^+^], 426.1140; found 426.1162. 

*2-(4-(Bis(benzyloxy)phosphoryl)butanamido)benzoic acid* (**12n**). Compound **12j** (0.416 g) was dissolved in a dichloromethane/TFA mixture (5/1, 8 mL) at 0 °C. After stirring for an hour, TLC analysis showed a completed reaction. Toluene (15 mL) was then added to the reaction mixture before concentration *in vacuo*. Column chromatography (97.5% CH_2_Cl_2_/2% MeOH/0.5% CH_3_COOH) yielded 272 mg of **12n** as an oil (73% yield). ^1^H-NMR (300 MHz, CDCl_3_) δ_H_ ppm 1.94–2.17 (m, 4H, P-CH_2_-CH_2_), 2.52 (t, *J* = 6.26 Hz, 2H, CH_2_-CONHPh), 4.87–5.14 (m, 4H, CH_2_-Ph), 7.06 (td, *J* = 1.08 Hz, 8.10 Hz, 1H, Ar-H), 7.27–7.35 (m, 10H, Ar-H), 7.51 (td, *J* = 1.08 Hz, 8.28 Hz, 1H, Ar-H), 8.10 (dd, *J* = 1.68 Hz, 8.10 Hz, 1H, Ar-H), 8.66 (td, *J* = 1.00 Hz, 8.39 Hz, 1H, Ar-H), 11.44 (br.s 1H, NH). ^13^C-NMR (75 MHz, CDCl_3_) δ_C_ ppm 18.25 (d, ^2^*J*_P-C_ = 5.04 Hz, C2), 25.22 (d, ^1^*J*_P-C_ = 140.04 Hz, C1), 38.52 (d, ^3^*J*_P-C_ = 17.22 Hz, C3), 67.96 (d, ^2^*J*_P-C_ = 6.52 Hz, PhCH_2_), 115.55 (Ar-C), 120.23 (Ar-C), 122.72 (Ar-C), 128.21 (Ar-C), 128.77 (Ar-C), 128.86 (Ar-C), 131.81 (Ar-C), 134.71 (Ar-C), 136.14 (d, ^3^*J*_P-C_ = 5.92 Hz, C_ipso_-PhCH_2_), 141.89 (Ar-C), 170.90 (CO), 170.98 (CO). ^31^P-NMR (121.5 MHz, CDCl_3_): δ_P_ ppm = 26.02.

*Diethyl 3-(2-cyanophenylcarbamoyl)propylphosphonate* (**13q**). ^1^H-NMR (300 MHz, CDCl_3_) δ_H_ ppm 1.34 (t, *J* = 7.11 Hz, 6H, P-O-CH_2_CH_3_), 1.79–2.17 (m, 4H, P-CH_2_-CH_2_), 2.64 (t, *J* = 7.11 Hz, 2H, CH_2_-CONHPh), 4.01–4.23 (m, 4H, -O-CH_2_-CH_3_), 7.19 (dd, *J* = 1.05 Hz, 7.64 Hz, 1H, Ar-H), 7.51–7.66 (m, 2H, Ar-H), 8.14 (br. s, 1H, NH), 8.28 (dd, *J* = 1.10 Hz, 8.96 Hz, 1H Ar-H). ^13^C-NMR (75 MHz, CDCl_3_) δ_C_ ppm 16.49 (d, ^3^*J*_P-C_ = 6.32 Hz, P-O-CH_2_-CH_3_), 18.58 (d, ^2^*J*_P-C_ = 6.32 Hz, C2), 24.43 (d, ^1^*J*_P-C_ = 141.21 Hz, C1), 37.02 (d, ^3^*J*_P-C_ = 12.19 Hz, C3), 61.75 (d, ^2^*J*_P-C_ = 6.06 Hz, P-O-CH_2_-CH_3_), 102.97 (Ar-C), 116.46 (CN), 122.10 (Ar-C), 124.39 (Ar-C), 132.46 (Ar-C), 134.04 (Ar-C), 140.37 (Ar-C), 170.86 (CO). ^31^P-NMR (121.5 MHz, CDCl_3_): δ_P_ ppm = 32.14. HRMS (ESI): calculated for C_25_H_22_N_2_O_4_P [(M+H)^+^], 325.1317; found 325.1317.

### 3.3. General Procedure for Amide Deprotection Yielding Targets **8a**–**i**, **m**–**p**

The amide (100–150 mg) was dissolved in MeOH (10 mL) and Pd/C (10%) was added under inert atmosphere. The resulting mixture was then stirred under hydrogen atmosphere for 10 min and the progress monitored by mass spectrometry. At completion, the reaction mixture was filtered and neutralized with 1 eq. of a NaOH. The mixture was concentrated *in vacuo*, re-dissolved in a mixture of water and *ter*-butanol, frozen and lyophilized to afford the desired targets compounds **8a**–**i**, **m**–**p** as a white powder in quantitative yield.

*Sodium hydrogen 3-(phenylcarbamoyl)propylphosphonate* (**8a**). ^1^H-NMR (300 MHz, D_2_O) δ_H_ ppm 1.40–1.56 (m, 2H, -CH_2_-), 1.78–1.93 (m, 2H, P-CH_2_-), 2.46 (t, *J* = 7.47 Hz, 2H, CH_2_-CONHPh), 7.24 (dt, *J* = 5.78, 2.82 Hz, 1H, Ar-H), 7.34–7.46 (m, 4 H, Ar-H). ^13^C-NMR (75 MHz, D_2_O) δ_C_ ppm 21.13 (d, ^2^*J*_P-C_ = 3.71 Hz, C2), 28.55 (d, ^1^*J*_P-C_ = 131.25 Hz, C1), 38.10 (d, ^3^*J*_P-C_ = 16.61 Hz, C3), 122.45 (Ar-C), 125.79 (Ar-C), 129.33 (Ar-C), 136.92 (Ar-C), 176.00 (CO). ^31^P-NMR (121.5 MHz, D_2_O): δ_P_ ppm = 22.06. HRMS (ESI): calculated for C_10_H_13_NO_4_P [(M−H)^−^], 242.0588; found 242.0061. 

*Sodium hydrogen 3-(o-tolylcarbamoyl)propylphosphonate* (**8b**). ^1^H-NMR (300 MHz, D_2_O) δ_H_ ppm 1.47–1.62 (m, 2H, -CH_2_-), 1.70–1.90 (m, 2H, P-CH_2_-), 2.10 (s, Ph-CH_3_), 2.40 (t, *J* = 7.44 Hz, 2H, CH_2_-CONHPh), 7.18–7.35 (m, 4H, Ar-H), 8.42 (br. s, 1H, NH). ^13^C-NMR (75 MHz, D_2_O) δ_C_ ppm 17.14 (Ph-CH_3_), 21.06 (d, ^2^*J*_P-C_ = 3.65 Hz, C2), 28.42 (d, ^1^*J*_P-C_ = 131.87 Hz, C1), 37.68 (d, ^3^*J*_P-C_ = 16.61 Hz, C3), 126.76 (Ar-C), 127.21 (Ar-C), 127.81 (Ar-C), 130.89 (Ar-C), 134.56 (Ar-C), 137.97 (Ar-C), 176.47 (CO). ^31^P-NMR (121.5 MHz, D_2_O): δ_P_ ppm = 23.60. HRMS (ESI): calculated for C_11_H_15_NO_4_P [(M−H)^−^], 256.0744; found 256.0322. 

*Sodium hydrogen 3-(2,6-dimethylphenylcarbamoyl)propylphosphonate* (**8c**). ^1^H-NMR (300 MHz, D_2_O) δ_H_ ppm 1.47–1.65 (m, 2H, -CH_2_-), 1.80–2.20 (m, 2H, P-CH_2_-), 2.17 (s, 6H, Ph-CH_3_), 2.54 (t, *J* = 7.47 Hz, 2H, CH_2_-CONHPh), 7.07–7.25 (m, 3H, Ar-H). ^13^C-NMR (75 MHz, D_2_O) δ_C_ ppm 17.44 (Ph-CH_3_), 21.11 (d, ^2^*J*_P-C_ = 3.44 Hz, C2), 28.68 (d, ^1^*J*_P-C_ = 131.75 Hz, C1), 37.24 (d, ^3^*J*_P-C_ = 17.28 Hz, C3), 128.21 (Ar-C), 133.48 (Ar-C), 136.31 (Ar-C), 176.37 (CO). ^31^P-NMR (121.5 MHz, D_2_O): δ_P_ ppm = 22.47. HRMS (ESI): calculated for C_12_H_17_NO_4_P [(M−H)^−^], 270.0901; found 270.0319. 

*Sodium hydrogen 3-(2-methoxyphenylcarbamoyl)propylphosphonate* (**8d**). ^1^H-NMR (300 MHz, D_2_O) δ_H_ ppm 1.37–1.52 (m, 2H, -CH_2_-), 1.77–1.92 (m, 2H, P-CH_2_-), 2.47 (t, *J* = 7.52 Hz, 2H, CH_2_-CONHPh), 3.83 (s, 3H, Ph-O-CH_3_), 7.00 (td, *J* = 7.65 Hz, 1.33 Hz, 1H, Ar-H), 7.09 (dd, *J* = 8.31 Hz, 1.24 Hz, 1H, Ar-H), 7.20–7.32 (m, 1H, Ar-H), 7.52 (dd, *J*= 7.87 Hz, 1.68 Hz, 1H, Ar-H). ^13^C-NMR (75 MHz, D_2_O) δ_C_ ppm 21.40 (d, ^2^*J*_P-C_ = 3.36 Hz, C2), 28.94 (d, ^1^*J*_P-C_ = 130.12 Hz, C1), 38.04 (d, ^3^*J*_P-C_ = 16.84 Hz, C3), 56.01 (-Ph-O-CH_3_), 112.30 (Ar-C), 121.07 (Ar-C), 125.20 (Ar-C), 125.53 (Ar-C), 127.65 (Ar-C), 152.22 (Ar-C), 176.45 (CO). ^31^P-NMR (121.5 MHz, D_2_O): δ_P_ ppm = 21.28. HRMS (ESI): calculated for C_11_H_15_NO_5_P [(M−H)^−^], 272.0693; found 272.0129.

*Sodium hydrogen 3-(2,6-dimethoxyphenylcarbamoyl)propylphosphonate* (**8e**). ^1^H-NMR (300 MHz, D_2_O) δ_H_ ppm 1.42–1.57 (m, 2H, -CH_2_-), 1.69–1.88 (m, 2H, P-CH_2_-), 2.40 (t, *J* = 7.39 Hz, 2H, CH_2_-CONHPh), 3.71 (s, 6H, Ph-O-CH_3_), 6.66 (d, *J* = 8.47 Hz, 2H, Ar-H), 7.33 (t, *J* = 8.47 Hz, 1H, Ar-H). ^13^C-NMR (75 MHz, D_2_O) δ_C_ ppm 20.82 (d, ^2^*J*_P-C_ = 3.64 Hz, C2), 28.05 (d, ^1^*J*_P-C_ = 132.26 Hz, C1), 37.12 (d, ^3^*J*_P-C_ = 17.22 Hz, C3), 56.34 (PhOCH_3_), 105.38 (Ar-C), 113.06 (Ar-C), 129.44 (Ar-C), 155.33 (Ar-C), 176.72 (CO). ^31^P-NMR (121.5 MHz, D_2_O): δ_P_ ppm = 24.34. HRMS (ESI): calculated for C_12_H_17_NO_6_P [(M+H)^+^], 302.0799; found 302.0074. 

*Sodium hydrogen 3-(2-fluorophenylcarbamoyl)propylphosphonate* (**8f**). ^1^H-NMR (300 MHz, D_2_O) δ_H_ ppm 1.44–1.61 (m, 2H, -CH_2_-), 1.80–1.94 (m, 2H, P-CH_2_-), 2.51 (t, *J* = 7.32 Hz, 2H, CH_2_-CONHPh), 7.13–7.33 (m, 3H, Ar-H), 7.53 (td, *J* = 1.74 Hz, 7.63 Hz, 1H, Ar-H). ^13^C-NMR (75 MHz, D_2_O) δ_C_ ppm 20.89 (d, ^2^*J*_P-C_ = 3.54 Hz, C2), 28.30 (d, ^1^*J*_P-C_ = 131.37 Hz, C1), 37.50 (d, ^3^*J*_P-C_ = 17.13 Hz, C3), 116.05 (d, *J*_F-C _= 19.91 Hz, Ar-C), 124.03 (d, *J*_F-C _= 3.36 Hz, Ar-C), 124.71 (d, *J*_F-C _= 12.74 Hz, Ar-C), 126.62 (Ar-C), 128.08 (d, *J*_F-C _= 7.95 Hz, Ar-C), 157.44 (Ar-C), 176.42 (CO). ^31^P-NMR (121.5 MHz, D_2_O): δ_P_ ppm = 22.63. HRMS (ESI): calculated for C_10_H_12_FNO_4_P [(M−H)^−^], 260.0494; found 260.0001.

*Sodium hydrogen 3-(2-acetylphenylcarbamoyl)propylphosphonate* (**8g**). ^1^H-NMR (300 MHz, D_2_O) δ_H_ ppm 1.24 (s, 3H, PhCOCH_3_), 1.40–1.58 (m, 2H, -CH_2_-), 1.84–1.99 (m, 2H, P-CH_2_-), 2.51 (t, *J* = 7.13 Hz, 2H, CH_2_-CONHPh), 7.20–7.43 (m, 4H, Ar-H), ^13^C-NMR (75 MHz, D_2_O) δ_C_ ppm 21.43 (d, ^2^*J*_P-C_ = 3.87 Hz, C2), 29.10 (d, ^1^*J*_P-C_ = 129.92 Hz, C1), 29.71 (PhCOCH_3_), 37.69 (d, ^3^*J*_P-C_ = 16.58 Hz, C3), 126.83 (Ar-C), 127.98 (Ar-C), 128.23 (Ar-C), 129.40 (Ar-C), 133.94 (Ar-C), 141.189 (Ar-C), 177.12 (-CO-), 177.20 (-COCH_3_). ^31^P-NMR (121.5 MHz, D_2_O): δ_P_ ppm = 22.19. HRMS (ESI): calculated for C_12_H_15_NO_5_P [(M−H)^−^], 284.0693; found 284.0693. 

*Sodium hydrogen 3-(2-(methylsulfonyl)phenylcarbamoyl)propylphosphonate* (**8h**). ^1^H-NMR (300 MHz, D_2_O) δ_H_ ppm 1.40–1.58 (m, 2H, -CH_2_-), 1.81–1.98 (m, 2H, P-CH_2_-), 2.57 (t, *J* = 7.66 Hz, 2H, CH_2_-CONHPh), 3.23 (s, 3H, -Ph-SO_2_CH_3_), 7.57 (td, *J* = 1.36 Hz, 7.73 Hz, 1H, Ar-H), 7.65 (dd, *J* = 1.36 Hz, 8.13 Hz, 1H, Ar-H), 7.79 (td, *J* = 1.49 Hz, 7.73 Hz, 1H, Ar-H), 8.01 (dd, *J* = 8.00 Hz, 1.53 Hz, 1H, Ar-H). ^13^C-NMR (75 MHz, D2O) δ_C_ ppm 20.97 (d, ^2^*J*_P-C_ = 3.37 Hz, C2), 28.89 (d, ^1^*J*_P-C_ = 130.92 Hz, C1), 37.91 (d, ^3^*J*_P-C_ = 17.13 Hz, C3), 43.14 (-Ph-SO_2_CH_3_), 128.31 (Ar-C), 129.60 (Ar-C), 129.83 (Ar-C), 133.81 (Ar-C), 134.67 (Ar-C), 135.82 (Ar-C), 177.16 (CO). ^31^P-NMR (121.5 MHz, D_2_O): δ_P_ ppm = 22.51. HRMS (ESI): calculated for C_11_H_15_NO_6_PS [(M−H)^−^], 320.0363; found 319.9703.

*Sodium hydrogen 3-(2-(dimethylamino)phenylcarbamoyl)propylphosphonate* (**8i**). ^1^H-NMR (300 MHz, D_2_O) δ_H_ ppm 1.37–1.56 (m, 2H, -CH_2_-), 1.79–1.96 (m, 2H, P-CH_2_-), 2.51 (t, *J* = 7.52 Hz, 2H, CH_2_-CONHPh), 2.62 (s, 6H, Ph-N-CH_3_), 7.07–7.15 (m, 1H, Ar-H), 7.22–7.29 (m, 2H, Ar-H), 7.45 (app. d, *J* = 7.65 Hz, 1H, Ar-H). ^13^C-NMR (75 MHz, D_2_O) δ_C_ ppm 21.31 (d, ^2^*J*_P-C_ = 3.69 Hz, C2), 29.11 (d, ^1^*J*_P-C_ = 130.27 Hz, C1), 38.17 (d, ^3^*J*_P-C_ = 16.91 Hz, C3), 43.71 (Ph-N-CH_3_), 120.11 (Ar-C), 123.95 (Ar-C), 126.90 (Ar-C), 127.79 (Ar-C), 129.99 (Ar-C), 147.99 (Ar-C), 176.57 (CO). ^31^P-NMR (121.5 MHz, D_2_O): δ_P_ ppm = 24.24. HRMS (ESI): calculated for C_12_H_18_N_2_O_4_P [(M−H)^−^], 285.1010; found 285.0459. 

*Sodium hydrogen (4-(methylsulfonamido)-4-oxobutyl)phosphonate* (**8m**). ^1^H-NMR (300 MHz, D_2_O) δ_H_ ppm 1.55–1.69 (m, 2H, -CH_2_-), 1.78–1.92 (m, 2H, P-CH_2_-), 2.40 (t, *J* = 7.27 Hz, 2H, CH_2_-CONHPh), 2.39 (s, 3H, -N-SO_2_CH_3_). ^13^C-NMR (75 MHz, D_2_O) δ_C_ ppm 19.78 (d, ^2^*J*_P-C_ = 3.87 Hz, C2), 27.41 (d, ^1^*J*_P-C_ = 133.24 Hz, C1), 38.62 (d, ^3^*J*_P-C_ = 17.14 Hz, C3), 40.10 (-N-SO_2_CH_3_), 180.33 (CO). ^31^P-NMR (121.5 MHz, D_2_O): δ_P_ ppm = 25.22. HRMS (ESI): calculated for C_5_H_11_NO_6_PS [(M−H)^−^], 244.0050; found 244.0611. 

*Sodium hydrogen 3-(2-carboxyphenylcarbamoyl)propylphosphonate* (**8n**). ^1^H-NMR (300 MHz, D_2_O) δ_H_ ppm 1.55–1.70 (m, 2H, -CH_2_-), 1.81–1.98 (m, 2H, P-CH_2_-), 2.51 (t, *J* = 7.31 Hz, 2H, CH_2_-CONHPh), 7.22 (td, *J* = 1.03 Hz, 7.64 Hz, 1H, Ar-H), 7.50 (td, *J* = 1.65 Hz, 7.64 Hz, 1H, Ar-H), 7.85 (dd, *J* = 7.83, 1.60 Hz, 1H, Ar-H), 8.01 (app. d, 1H, Ar-H). ^13^C-NMR (75 MHz, D_2_O) δ_C_ ppm 19.97 (d, ^2^*J*_P-C_ = 3.95 Hz, C2), 27.37 (d, ^1^*J*_P-C_ = 133.39 Hz, C1), 38.45 (d, ^3^*J*_P-C_ = 17.66 Hz, C3), 121.99 (Ar-C), 124.67 (Ar-C), 125.10 (Ar-C), 130.71 (Ar-C), 132.22 (Ar-C), 137.17 (Ar-C), 173.83 (CO, PhCOOH), 174.88 (CO, -CH_2_-CO-NH-). ^31^P-NMR (121.5 MHz, D_2_O): δ_P_ ppm = 24.98. HRMS (ESI): calculated for C_11_H_13_NO_6_P [(M−H)^−^], 286.0486; found 286.0268.

*Sodium hydrogen 3-(2-hydroxyphenylcarbamoyl)propylphosphonate* (**8o**). ^1^H-NMR (300 MHz, D_2_O) δ_H_ ppm 1.46–1.61 (m, 2H, -CH_2_-), 1.79–1.96 (m, 2H, P-CH_2_-), 2.51 (t, *J* = 7.44 Hz, 2H, CH_2_-CONHPh), 6.88–7.03 (m, 2H, Ar-H), 7.18 (td, *J* = 1.79 Hz, 7.45 Hz, 1H, Ar-H), 7.35 (dd, *J* = 7.83 Hz, 1.60 Hz, 1H, Ar-H). ^13^C-NMR (75 MHz, D_2_O) δ_C_ ppm 20.85 (d, ^2^*J*_P-C_ = 3.84 Hz, C2), 28.25 (d, ^1^*J*_P-C_ = 131.65 Hz, C1), 37.47 (d, ^3^*J*_P-C_ = 17.18 Hz, C3), 116.89 (Ar-C), 120.73 (Ar-C), 124.10 (Ar-C), 126.37 (Ar-C), 128.15 (Ar-C), 149.83 (Ar-C), 176.39 (CO). ^31^P-NMR (121.5 MHz, D_2_O): δ_P_ ppm = 22.85. HRMS (ESI): calculated for C_10_H_13_NO_5_P [(M+H)^+^], 258.0537; found 258.0058. 

*Sodium hydrogen 3-(2,6-dihydroxyphenylcarbamoyl)propylphosphonate* (**8p**). ^1^H-NMR (300 MHz, D_2_O) δ_H_ ppm 1.48–1.66 (m, 2H, -CH_2_-), 1.78–1.99 (m, 2H, P-CH_2_-), 2.54 (t, *J* = 7.43 Hz, 2H, CH_2_-CONHPh), 6.52 (d, *J* = 8.33 Hz, 2H, Ar-H), 7.07 (t, *J* = 8.22 Hz, 1H, Ar-H). ^13^C-NMR (75 MHz, D_2_O) δ_C_ ppm 20.63 (d, ^2^*J*_P-C_ = 3.95 Hz, C2), 28.12 (d, ^1^*J*_P-C_ = 131.80 Hz, C1), 37.02 (d, ^3^*J*_P-C_ = 16.52 Hz, C3), 108.25 (Ar-C), 111.93 (Ar-C), 129.19 (Ar-C), 152.84 (Ar-C), 177.01 (CO). ^31^P-NMR (121.5 MHz, D_2_O): δ_P_ ppm = 22.22. HRMS (ESI): calculated for C_10_H_13_NO_6_P [(M−H)^−^], 274.0486; found 273.9962. 

*Bisammomium 3-(2-cyanophenylcarbamoyl)propylphosphonate* (**8q**). Intermediate **13q** (150 mg, 0.334 mmol) was dissolved in dry dichloromethane (6 mL) under inert atmosphere and cooled to 0 °C. TMSBr (0.5 mL, 3.3 mmol) was added dropwise while stirring. The icebath was removed after 10 min and the reaction stirred at room temperature for 24 h. ^31^P-NMR confirmed that the starting phosphonate was completely deprotected (shift from δ = 32–25 ppm). The volatiles were removed *in vacuo*, the crude material was dissolved in 5% aqueous ammonia and washed with diethyl ether. Lyophilisation of the ammonia solution yielded the product as a brown solid in quantitative yield. ^1^H-NMR (300 MHz, D_2_O) δ_H_ ppm 1.50–1.65 (m, 2H, -CH_2_-), 1.85–2.20 (m, 2H, P-CH_2_-), 2.68 (t, *J* = 7.58 Hz, 2H, CH_2_-CONHPh), 7.45 (td, *J* = 0.99 Hz, 7.96 Hz, 1H, Ar-H), 7.60 (d, *J* = 8.05 Hz, 1H, Ar-H), 7.77 (td, *J* = 1.51 Hz, 7.20 Hz, 1H, Ar-H), 8.08 (dd, *J* = 1.33 Hz, 7.96 Hz, 1H, Ar-H). ^13^C-NMR (75 MHz, D_2_O) δ_C_ ppm 21.55 (d, ^2^*J*_P-C_ = 3.87 Hz, C2), 27.80 (d, ^1^*J*_P-C_ = 136.00 Hz, C1), 35.65 (d, ^3^*J*_P-C_ = 16.59 Hz, C3), 121.59 (Ph-CN), 126.40 (Ar-C), 126.65 (Ar-C), 127.45 (Ar-C), 134.95 (Ar-C), 149.45 (Ar-C), 157.68 (Ar-C), 162.45 (CO). ^31^P-NMR (121.5 MHz, D_2_O): δ_P_ ppm = 25.00. HRMS (ESI): calculated for C_11_H_13_N_2_O_4_P [(M−H)^−^], 267.0540; found 267.0823.

### 3.4. Synthesis of o-(Dimethylamino)aniline (**11i**)

To a solution of **14** (0.5 g; 2 mmol) in MeOH (100 mL) was added formalin (14 mL), Pd/C 10% (160 mg) and formic acid (1 mL). The resulting mixture was allowed to stir under a hydrogen atmosphere for 3 h, after which, the mixture was filtered over a celite path and the filtrate concentrated to about 25 mL. The mixture was then basified by adding NaHCO_3_ and the water layer was extracted three times with EtOAc (3 × 50 mL). The combined organic phase was washed once with brine and dried over Na_2_SO_4_. Column chromatography (hexane/EtOAc 95:5) yielded **15** (0.450 g, 90%) as a colorless oil. Subsequent treatment of **15** with 30% TFA in dichloromethane at 0 °C afforded **11i** which was used for the next step without further purification.

*tert-Butyl 2-(dimethylamino)phenylcarbamate* (**15**). ^1^H-NMR (300 MHz, CDCl_3_) δ_H_ ppm 1.54 (br. s, 9H, *tert*-Bu), 2.62 (s, 6H, N-CH_3_), 6.96 (td, *J* = 1.16 Hz, 7.57 Hz, 1H, Ar-H), 7.05–7.16 (m, 2H, Ar-H), 7.70 (br. s, 1H, NH), 8.07 (d, *J* = 8.17). ^13^C-NMR (75 MHz, CDCl_3_) δ_C_ ppm 28.93 (CH_3_ of *tert*-Bu), 44.83 (N-CH_3_), 80.27 (C_q_ of *tert*-Bu), 117.97 (Ar-C), 120.16 (Ar-C), 122.51 (Ar-C), 125.22 (Ar-C), 134.13 (Ar-C), 142.35 (Ar-C), 153.29 (CO). HRMS (ESI): calculated for C_13_H_21_N_2_O_2_ [(M+H)^+^], 237.1598; found 237.1602. 

## 4. Conclusions

In conclusion, amide derivatives of fosmidomycin were synthesized from simple starting materials. These analogues were inactive against *E. coli Dxr*, Mtb Dxr and *P. falciparum* K1 possibly due their inability to adopt a favorable conformation necessary for the Dxr active site metal chelation. Replacing the hydroxamate group of fosmidomycin with an alternative and efficient bidentate metal binding group in Dxr inhibitors, remains a daunting challenge as previously noted [36]. 
